# Transcriptome and iTRAQ-Based Proteome Reveal the Molecular Mechanism of Intestinal Injury Induced by Weaning Ewe's Milk in Lambs

**DOI:** 10.3389/fvets.2022.809188

**Published:** 2022-04-25

**Authors:** Lulu Han, Hui Tao, Lingyun Kang, Shuo Wang, Qiyu Diao, Deping Han, Kai Cui

**Affiliations:** ^1^Key Laboratory of Feed Biotechnology of the Ministry of Agriculture and Rural Affairs, Institute of Feed Research of Chinese Academy of Agricultural Sciences, Beijing, China; ^2^State Key Laboratory of Animal Nutrition, College of Animal Science and Technology, China Agricultural University, Beijing, China; ^3^College of Veterinary Science, China Agricultural University, Beijing, China

**Keywords:** sheep, intestinal injury, weaning stress, transcriptome, proteome

## Abstract

Early feeding regime has a substantial lifelong effect on lambs and weaning ewe's milk can lead to the intestinal injury of lambs. To explore the molecular regulatory mechanism of intestinal injury of lambs under weaning stress, the jejunum was conducted transcriptome and then integrated analyzed with our previous proteome data. A total of 255 upregulated genes and 285 downregulated genes were significantly identified. These genes showed low overlapping with differentially expressed proteins identified by isobaric tags for relative and absolute quantification (iTRAQ). However, according to their functions, the differentially expressed genes (DEGs) and proteins with the same expression trend were enriched for the similar Gene Ontology (GO) terms and the Kyoto Encyclopedia of Genes and Genomes (KEGG) pathways, such as intestinal lipid absorption, urea cycle, peroxisome proliferator-activated receptor (PPAR) signaling pathway, and ferroptosis. Furthermore, the DEGs, including *FABP2, ACSL3, APOA2, APOC3*, and *PCK1*, might play essential roles in intestinal lipid absorption and immune response through the PPAR signaling pathway and ferroptosis. This study could provide new insights into early lamb breeding at the molecular level.

## Introduction

In the recent years, house feeding and intensive breeding have become the primary trend of the sheep industry due to the limitations of resources and the environment. To improve the utilization rate of ewes, the implementation of an artificial milk replacer feeding program to substitute ewe lactation has become a common practice of lamb breeding in many countries (1, 2). However, weaning is the most severe stimulating factor for the animals during early development, which is shown as the decreased feed intake, low nutrition absorption, aberrant mucosal immunity, and higher mortality (3–5). Young ruminants' intestinal histology and function would significantly change after the colonization of residential microorganisms, invasion of exogenous pathogens, and deletion of maternal antibodies (6–8), closely related to their growth, inflammatory response, and susceptibility to some diseases (5).

The small intestine, a critical multifunctional organ, plays crucial roles in digesting and absorbing nutrients, regulating immune response, and secreting various enzymes and immunoglobulins (8, 9). The intestinal mucosa is the largest interface between the host and the external environment, so intestinal cells are continuously exposed to the coexistence of symbiotic bacteria, diets, pathogenic microorganisms, and metabolites. For this reason, the intestinal epithelium and lamina propria form a multilayer and complex barrier mechanism to maintain intestinal homeostasis. However, the structure and function of the intestine are affected by weaning, which is essential for lambs whose digestive and immune systems are not yet mature (10). With the intestinal microflora disturbance, the metabolic imbalance, and the inflammatory reaction induced by pathogen invasion after weaning, the homeostasis of the intestine is broken. Therefore, it is urgent to understand the molecular mechanism of lamb intestinal injury caused by weaning ewe's milk, which could help us to draw better methods to minimize this effect.

Due to the high throughput and quantifiable advantage, RNA sequencing (RNA-seq) could detect the bulk differentially expressed genes (DEGs) (11), widely used to identify gene expressions and modulated pathways. Therefore, in this study, RNA-seq was performed to analyze the transcriptome of lamb jejunum. After screening the differentially expressed genes, we annotated their function to investigate the regulation of intestinal injury under weaning stress. At the same time, our previous proteomic data were integrated with the transcriptomic data to determine the same Gene Ontology (GO) terms and the Kyoto Encyclopedia of Genes and Genomes (KEGG) pathway items enriched in the two data sets. Those results provide more basis for exploring the molecular mechanism of lamb intestinal injury induced by weaning ewe's milk, which could help us to better understand intestinal function regulation after weaning.

## Materials and Methods

### Animals, Experimental Design, and Sampling

The same animals were used according to our previous study (6). In summary, eight pairs of twin neonatal ram Hu lambs born on the same day with almost the same body weight (2.75 ± 0.2 kg) were randomly divided into the two groups. One group was artificially reared (AR) with milk replacer fed four times a day after sucking colostrum for 3 days and the other group was ewe-reared (ER) during the entire 15 days experiment. Each pair of twin samples could be monozygotic and dizygotic twins and siblings to reduce the influence of inherent genes in exploration of the differences of gene expression after AR or ER feeding procedures. The commercial milk replacer was acquired from Precision Animal Nutrition Research Center (Beijing, China; CP: 25.08%, DE: 18.38%, EE: 11.18%, Ash: 5.29%, Ca: 1.13%, and P: 0.51% of DM) and the feed amount of milk replacer was adjusted in direct accordance with 2% of the lamb's body weight.

On 15 days of the experiment, three pairs of healthy twins with similar weight and same gender were slaughtered with a compressed air pistol to cause a cerebral concussion, followed by exsanguination by cutting the jugular and carotid veins. After slaughter, the middle part of the jejunum with 2 cm was sampled quickly and frozen in liquid nitrogen.

### Ribonucleic Acid Extraction, Sequencing, and Read Quality Control

The frozen jejunum samples were ground with liquid nitrogen and then weighted 1.0 g for each sample. Total RNA of each jejunum was extracted and purified using the Total RNA Extraction Kit (Invitrogen, Carlsbad, California, USA), according to the manufacturer's standard protocol. RNA integrity number (RIN) and accurate yield were initially measured by spectrophotometry on the Nanodrop 1000 (Nanodrop Technologies, Wilmington, Delaware, USA). Only high-quality RNAs (RIN > 8 and RNA yield > 2 μg) could be advanced to the next step. Individual complementary DNA (cDNA) libraries were generated from 0.5 μg of total RNA per sample using the Illumina TruSeq RNA Library Preparation Kit version 2 (Illumina Incorporation, San Diego, California, USA). Then, sequencing libraries were sequenced on the Illumina HiSeq 2500 (Illumina Incorporation) and generated 100 bp paired-end reads.

The raw reads were filtered and trimmed using Trimmomatic software (12) to remove low-quality reads and adapter sequences. In other words, the reads whose number of low-quality (sQ ≤ 5) bases contained in a single-ended sequencing read exceeded 50% of the total number of bases and N content > 10% were removed. The generated clean reads were aligned against sheep genome reference sequences (OARv4.0) by applying STAR aligner (13) with default parameters. Mapped reads were sorted by the Sequence Alignment/Map tools (SAM tools) (14) and then counted by gene using featureCounts (15) embedded in Subread (16).

### Statistical Analysis

Gene expression levels in each library were normalized to fragments per kilobase of exon per million mapped reads (FPKM). The significant DEGs in pairwise comparison between the AR and ER groups were determined using the DESeq2 R package (17). The selected model for identifying the DEGs incorporated group and maternal effect (design = ~ group effect + maternal effect). The maternal effect is mainly related to the lamb individuals and were collected from different ewes, which could infect the performance during lactation. Wald inferences tests were used to assign false discovery rate (FDR)-adjusted *p*-values (*q*-values) for each gene in each pairwise contrast. Both the significance level (*q*-value < 0.05) and magnitude of expression change [|log2(fold-change)| ≥ 1] were used to select the significant DEGs. The functional analysis, including the KEGG and GO, for DEGs was used ClueGo (version 2.8.5) in Cytoscape (version 3.7.2) (18). All the pathways were shown with a *q*-value < 0.05 and the kappa score was used to define term–term interrelation and the functional groups based on shared genes between the terms. Meanwhile, the network specificity was set to “medium.” Finally, STRING version 11 (http://string-db.org/) was used to predict the protein–protein interactions (PPIs) of DEGs. A PPI network was drawn using Cytoscape and the cytoHubba application was used to identify the top 10 hub genes (19, 20). The isobaric tags for relative and absolute quantification (iTRAQ) data of the same samples has been published in the previous study (6). In this integrative study, DEGs were identified by RNA-seq. In addition, the differentially expressed proteins (DEPs) from iTRAQ were compared and overlapped for consistent results. In brief, direct and indirect correlations were combined to get a complete relationship between the two omics. The specific operation methods are as follows: geninfo identifier (GI) of the proteome is mapped to gene symbol through the gene2accession in the National Center for Biotechnology Information (NCBI) and then compared with the gene number of the transcriptome. Then, for the unrelated genes, the database (transcript sequence of transcriptome) and query (identified protein sequence) were used for the Basic Local Alignment Search Tool (BLAST) sequence alignment, the e-value threshold was selected as 1e−5, and the identity (sequence similarity) was guaranteed to be more than 95%. Next, the Pearson correlation method was used to calculate the correlation of sample pair ratio in transcriptome and proteome. To show the correlation between two omics in detail, we further classified the DEGs revealed by RNA-seq and the DEPs identified by the previous report minutely *via* the threshold of expression difference: fold-change ≥ 1.5 in transcriptome and fold-change ≥ 1.2 in the proteome.

## Results

### Summary of Transcriptome Data

RNA-seq produced more than 18,195,848 raw reads (Table 1). After data filtering, 18,136,753–27,932,670 clean reads were generated to quantify transcripts and single nucleotide polymorphism (SNP) calling. The Guanine and cytosine (GC) content in the libraries ranged from 48.24 to 50.56%. The six samples had at least 91.91% reads with ≥Q20 and 85.81% reads with ≥Q30. The majority of reads in each library were mapped to the sheep genome reference sequences and the average mapping rates were 78.96 and 77.15% for the AR and ER groups, respectively. A total of 18,590 transcripts and 396,743 SNPs were identified in the samples.

**Table 1:** The quality of data output.

**Sample**	**Raw reads**	**Clean reads**	**Raw bases (G)**	**Clean bases (G)**	**Error (%)**	**Q20 (%)**	**Q30 (%)**	**GC (%)**
AR1	22995633	22373634	5.75	5.59	0.06	91.96	85.92	50.48
AR2	28353082	27932670	7.09	6.98	0.04	94.19	89.22	50.56
AR3	21275181	21220183	5.32	5.31	0.06	92.59	86.52	50.05
ER1	23576166	23185535	5.89	5.8	0.04	94.19	89.28	49.38
ER2	18195848	18136753	4.55	4.53	0.04	96.15	92.00	48.24
ER3	21778235	21267590	5.44	5.32	0.06	91.91	85.81	49.13

*Raw reads: the original sequence data was counted, four behaviors per unit, statistics of the number of sequencing sequences of each file*.

*Clean reads: the calculation method was the same as raw reads, but the statistical files were filtered sequencing data*.

*Raw bases (G): the number of original sequence data was multiplied by the length of the sequencing sequence, unit: G (data)*.

*Clean bases (G): the number of filtered sequences multiplied by the length of the sequence, unit: G (data)*.

*Error (%): base error rate. Q20, Q30 (%): the percentage of bases with Phred values > 20 and 30 to the total bases*.

*GC (%): the percentage of the total number of bases G and C to the total number of bases*.

**Figure 1:**
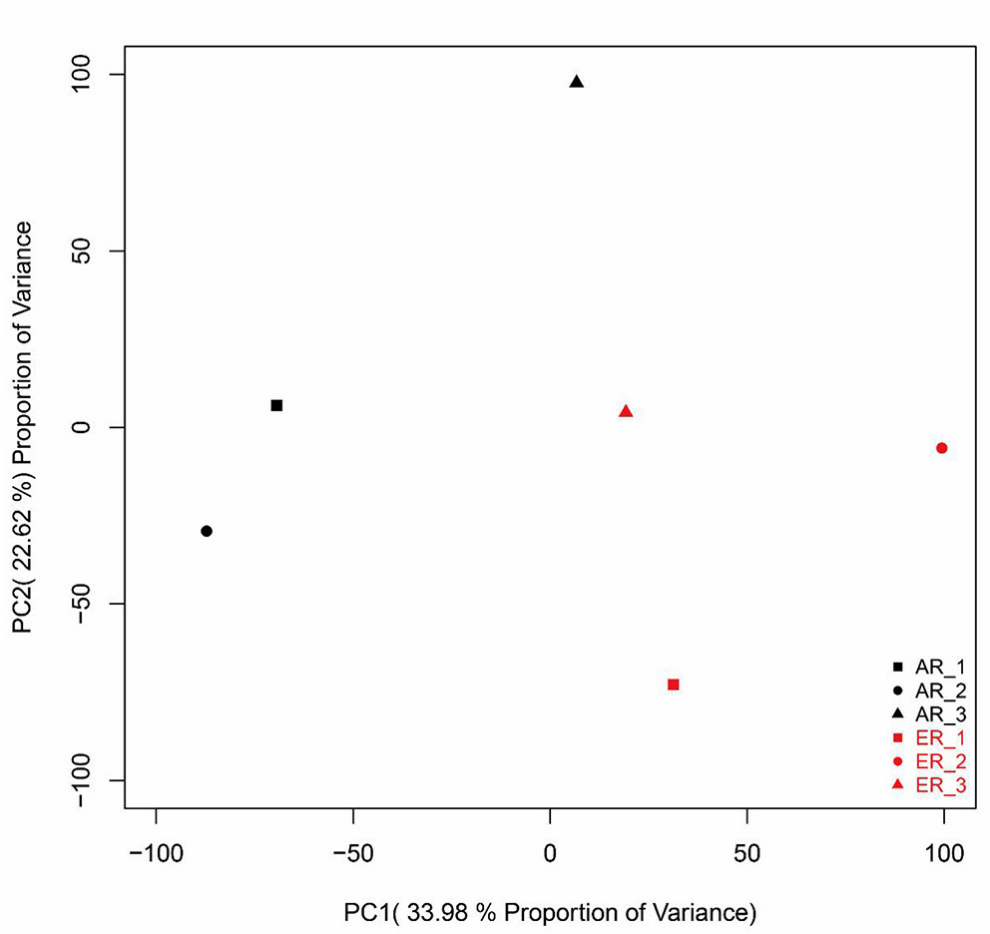
The principal component analysis (PCA) analysis of gene expressions in the intestinal samples. The black patterns represent the artificially reared (AR) group and the red patterns represent the ewe reared (ER) group.

The principal component analysis (PCA) of all the genes from intestinal samples showed separations between the two groups (Figure 1). Besides, samples were significantly divided into the two groups as experimental design (Supplementary Figure S1A). They were clustered into three branches as their ewe origination (Supplementary Figure S1B).

### Function of Differentially Expressed Genes

**Figure 2:**
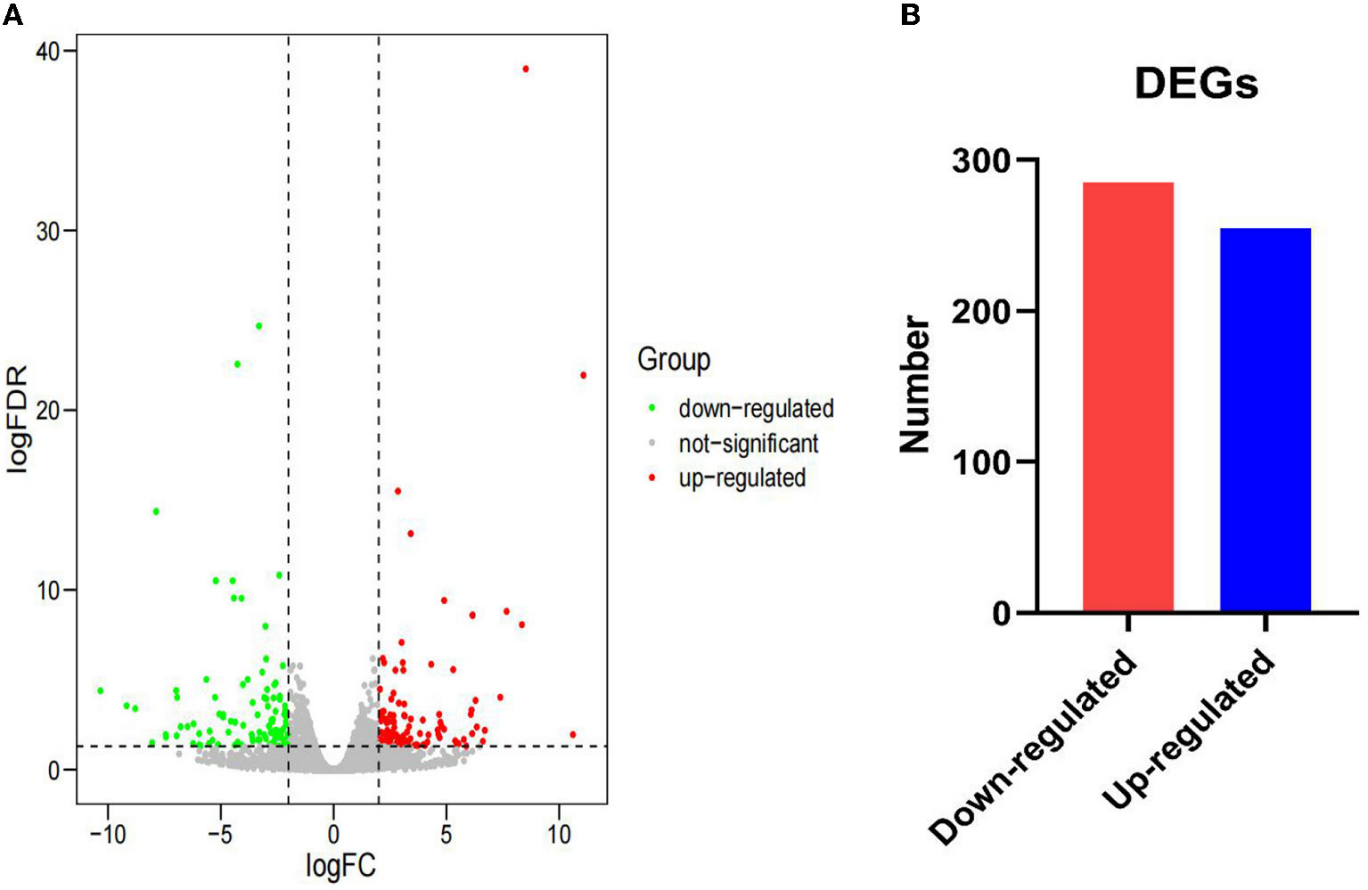
Gene expression between the AR and ER groups. **(A)** The expression of differentially expressed genes (DEGs) between the two groups. The red dots indicate upregulated DEGs and the green dots indicate downregulated DEGs. The gray dots reveal that there is no difference in the expression of genes between the two groups. **(B)** Number of significantly DEGs.

**Figure 3:**
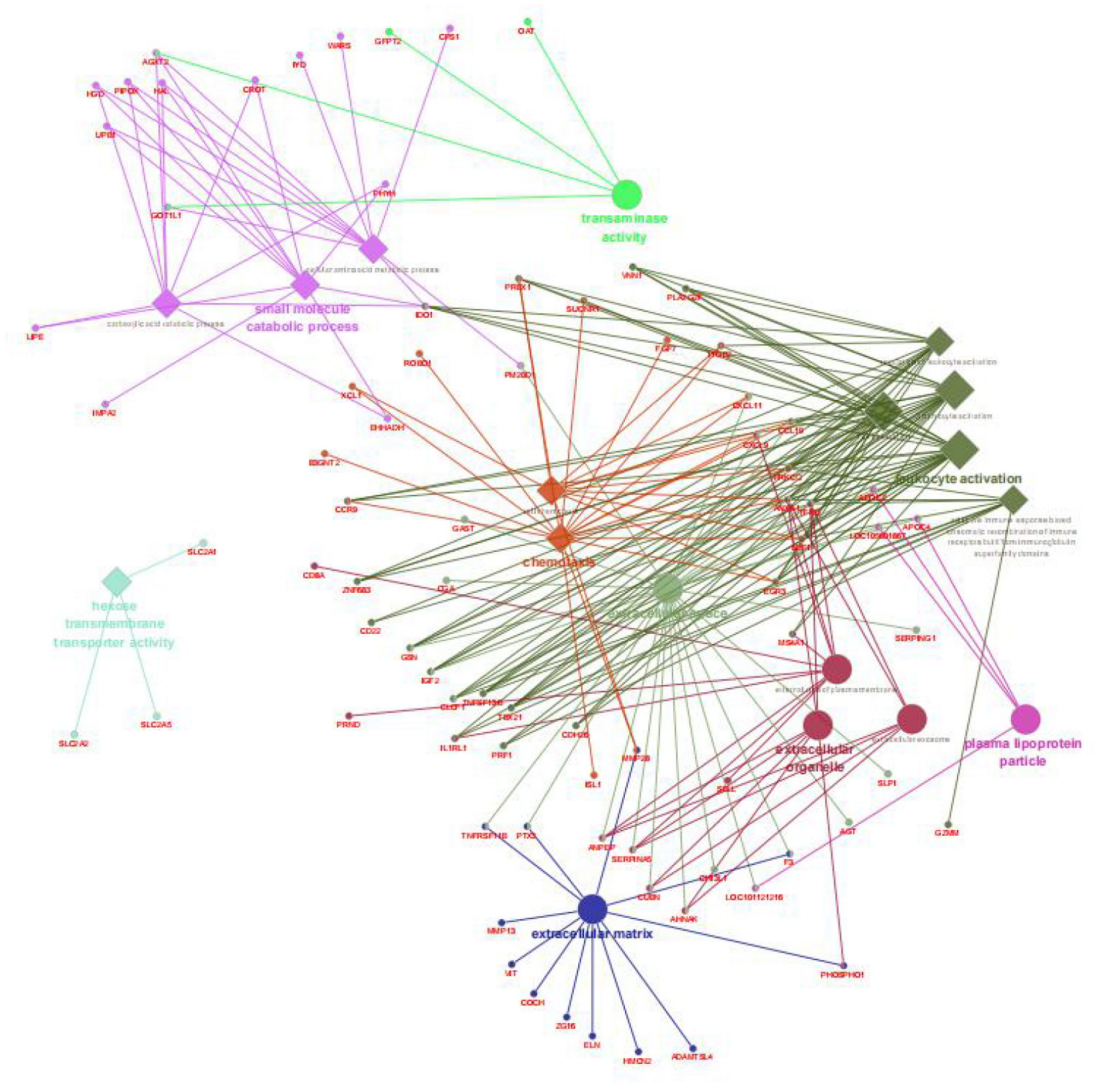
Enriched biological processes, cellular components, and molecular functions assigned to the DEGs between the AR and ER groups. The diamond showed the biological processes; the big circle showed the cellular components; the small circle showed the genes; the different colors represented the different functional groups, respectively, based on kappa score; all the DEGs were not enriched molecular functions.

After annotation, 255 upregulated DEGs and 285 downregulated DEGs in the AR group were compared with the ER group (Figures 2A,B). In this study, the genes related to lipid metabolic processes, defense response, regulation of immune response, and intestinal epithelium morphology verified by quantitative reverse transcription-PCR (qRT-PCR) in the proteome were consistent with their expression trends in the previous study (6). The GO enrichment analysis was carried out to explore the biological mechanisms underlying the two different feeding regimes. The 9 GO terms were enriched, including five biological processes (BPs) and four cellular components (CCs) on the DEGs (Figure 3). The significantly enriched BP terms were mainly related to the small molecule catabolic process, hexose transmembrane transporter activity, chemotaxis, and leukocyte activation. The enriched CC terms considerably were primarily involved in transaminase activity, extracellular space, extracellular matrix, extracellular organelle, and plasma lipoprotein particle.

**Figure 4:**
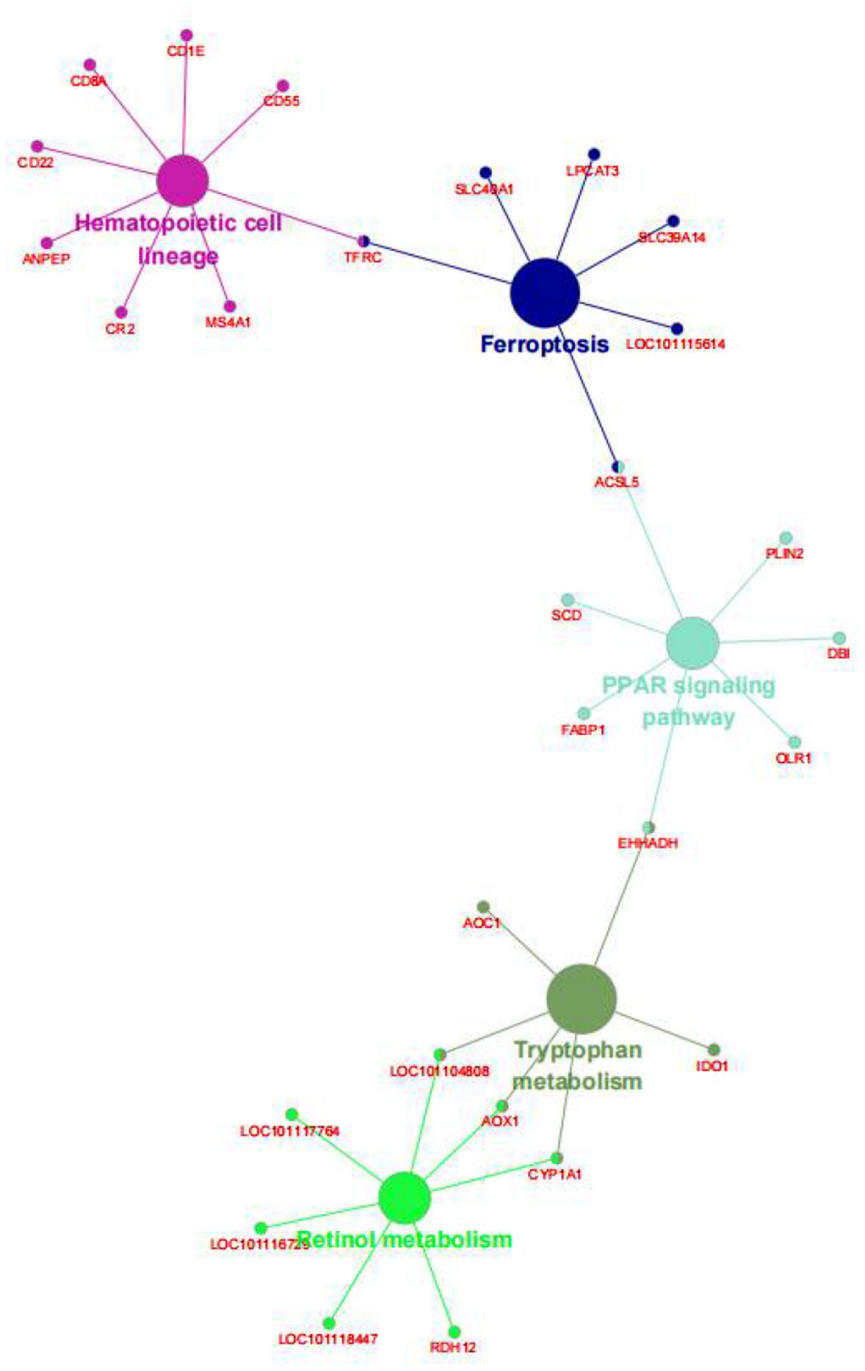
The Kyoto Encyclopedia of Genes and Genomes (KEGG) enrichment analysis of the DEGs between the AR and ER groups. The big circle showed the metabolic pathway; the small circle showed the genes; the different colors represented different pathways, respectively.

**Figure 5:**
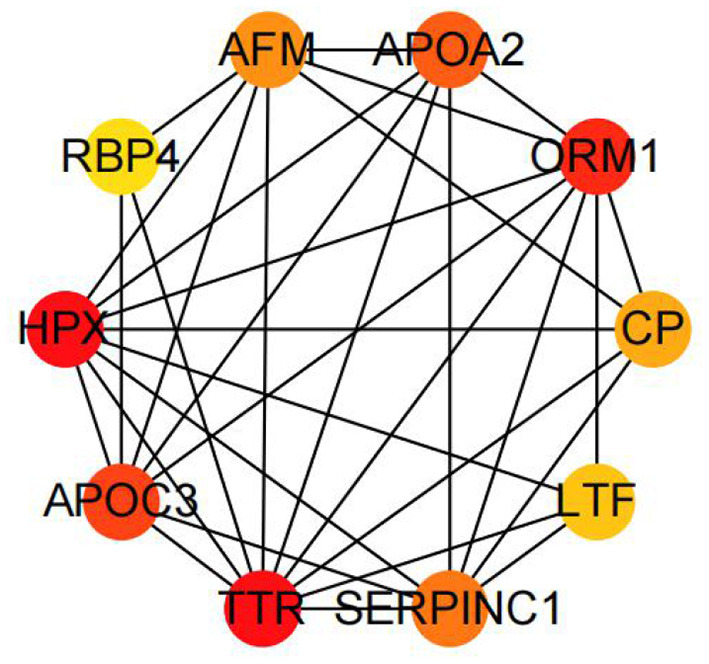
The top 10 hub genes by cytoHubba in the DEGs between the AR and ER groups.

We also performed the same enrichment analysis based on the KEGG pathway database and identified five pathways overrepresented in the pathway annotations of the given DEGs. As shown in Figure 4, the significant enrichment pathways were mainly associated with the peroxisome proliferator-activated receptor (PPAR) signaling pathway (*ACSL5, FABP1, EHHADH, OLR1, DBI, PLIN2*, and *SCD*), hematopoietic cell lineage (*CD8A, CD1E, CD22, CD55, TFRC, MS4A1, CR2*, and *ANPEP*), ferroptosis (*TFRC, SLC40A1, LPCAT3, 9LC39A14, LOC101115614*, and *ACSL5*), retinol metabolism (*LOC101104808, AOX1, CYP1A1, RDH12, LOC101118447, LOC101116729*, and *LOC101117764*), and tryptophan metabolism (*EHHADH, IDO1, CYP1A1, AOX1, LOC101104808*, and *AOC1*). Furthermore, we identified the top 10 hub genes among the DEGs, including *ORM1, HPX, TTR, APOA2, APOA3, CP, AFM, SERPINC1, LTF*, and *RBP4*, using the cytoHubba application (Figure 5).

### Correlation Analysis of Differentially Expressed Genes and the Previously Reported Differentially Expressed Proteins

**Figure 6:**
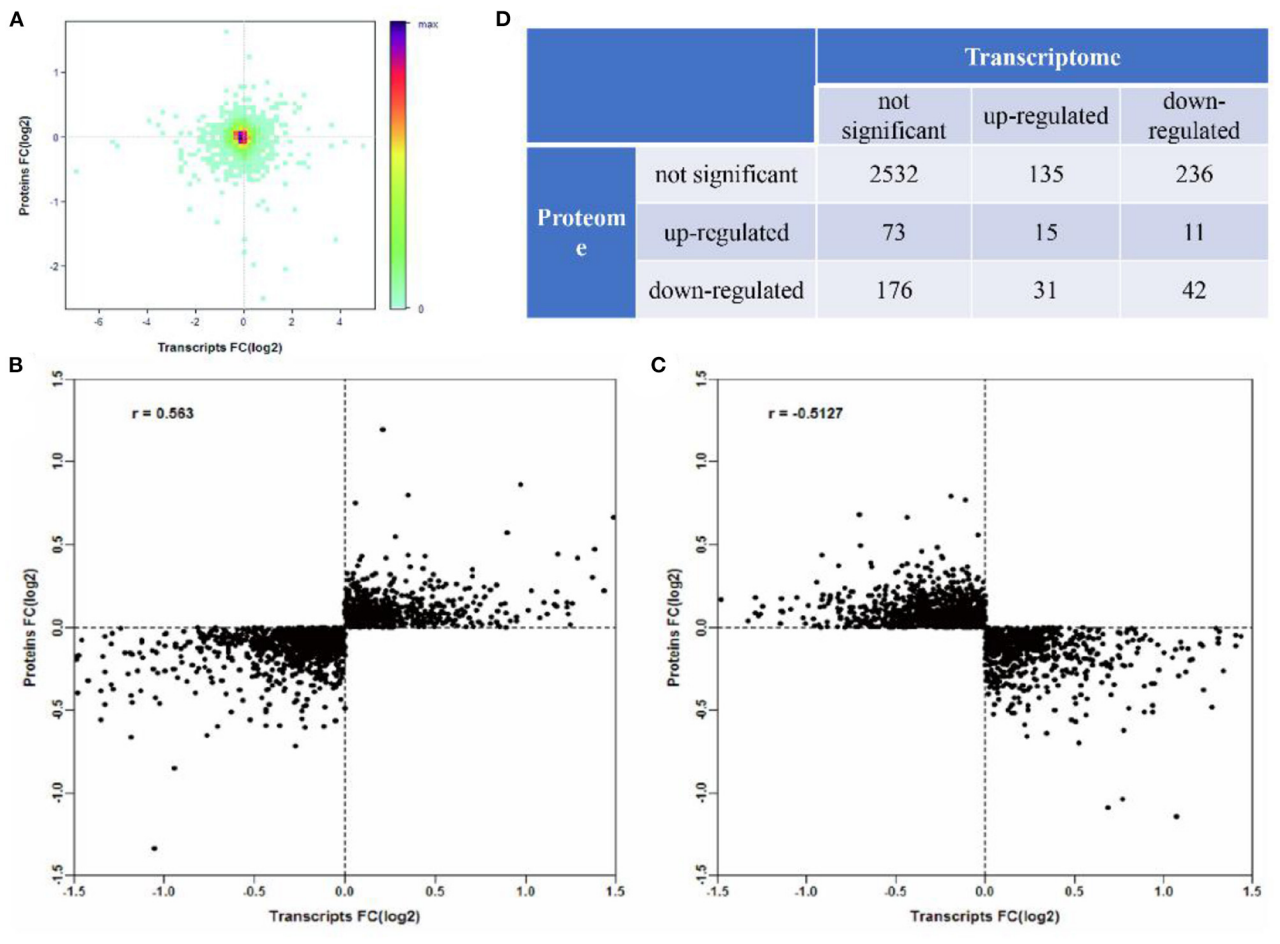
Differential correlation of proteome-transcriptome expression. **(A)** The correlation between all the associated protein and messenger RNA (mRNA). **(B)** The distribution of correlation of consistent-changed transcriptome and proteome. **(C)** The distribution of correlation of different-changed transcriptome and proteome. **(D)** Detailed classification of expression correlation.

**Figure 7:**
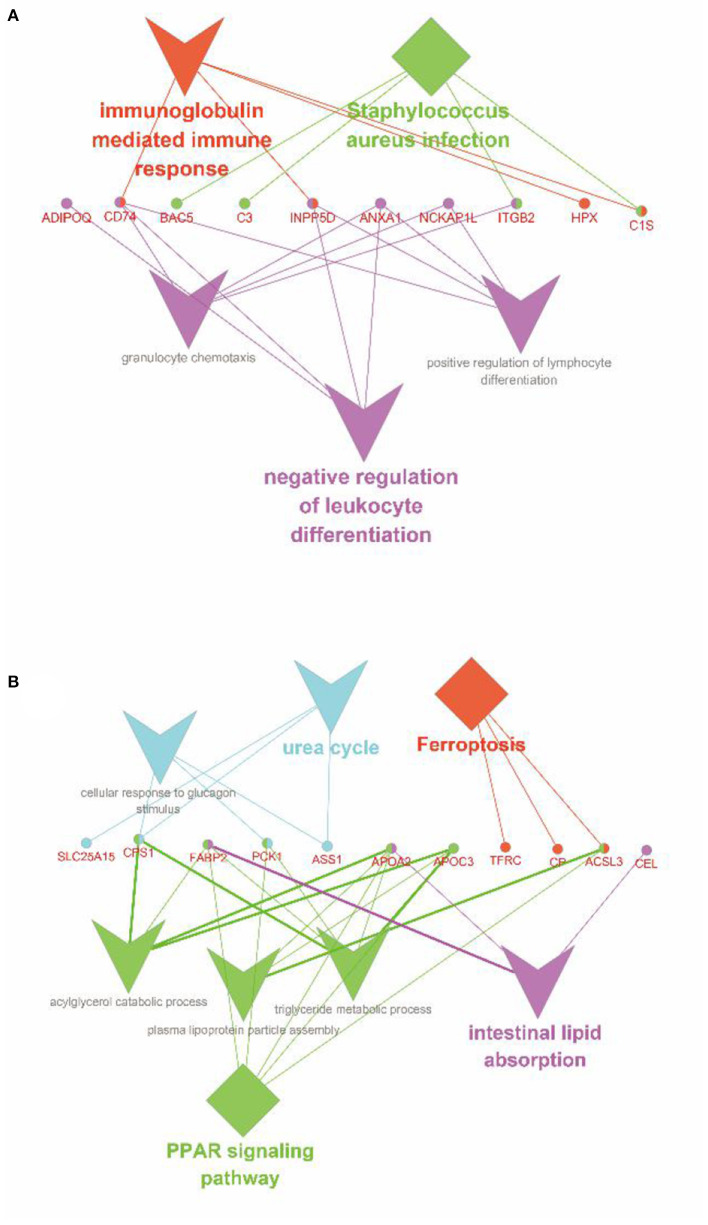
Enrichment analysis of the co-expressed DEGs between transcriptome and proteome. **(A)** Enrichment analysis of the genes, which were upregulated in transcriptome but downregulated in proteome. **(B)** Enrichment analysis of the genes, which were downregulated in transcriptome and proteome. The diamond represented the metabolic pathways in the KEGG database; the triangle represented the functions in the GO database; the circle showed the genes; the different colors represented the different functional groups, respectively, based on kappa score.

According to Pearson's analysis of the overall correlation between proteome and transcriptome expression changes, we found that transcriptome and proteome had a low correlation (Figure 6A). Moreover, the results of most genes are concentrated near the coordinate center 0, indicating that the expression of most genes at the transcriptome and proteome levels changes the same. There is no significant change. However, the correlation was significantly higher when the expression variation was consistent and opposite (Figures 6B,C). Further, the specific expression correlation between transcriptome and proteome is given in Figure 6D. Based on this, we analyzed the screened DEGs using the KEGG and the GO databases in GlueGo, respectively (Figures 7A,B). Interestingly, the genes that were upregulated in both the transcriptome and proteome or upregulated in transcriptome but downregulated in the proteome could not be enriched in the KEGG and the GO databases. Nevertheless, genes that were upregulated in transcriptome but downregulated in proteome were increased in the functions of immunoglobulin-mediated immune response, *Staphylococcus aureus* infection, and negative regulation of leukocyte differentiation. Meanwhile, the genes having the same downregulated trend in transcriptome and proteome were enriched in the PPAR signaling pathway (*APOA2, APOC3, ACSL3, CPS1, FABP2*, and *PCK1*), intestinal lipid absorption (*APOA2, FABP2*, and *CEL*), urea cycle (*CPS1, PCK1, SLC25A15*, and *ASS1*), and ferroptosis (*ACLS3, CP*, and *TFRC*).

## Discussion

The intestine is the largest immune and digestive organ of mammals, suffering from any damage, which will lead to a series of health problems. Its mucous membrane is the largest interface between the body and the external environment, digesting and metabolizing nutrients, and resisting the invasion of foreign substances. Therefore, in this study, twin samples from three ewes were selected to explore the effects of AR and ER feeding regimes on the intestine health of lambs. Early intestine tissue sections have shown that the stress of separation from the dam at weaning destroyed the inherent morphological structure of the intestine, resulting in intestinal villus atrophy and crypt depth deepening (6). To further explore the intestinal dysfunction of lambs caused by the stress of weaning ewe's milk, we carried out the transcriptome analysis of intestinal tissues of lambs after AR or ER feeding procedures by RNA-seq technique and the DEGs were screened. Based on the comprehensive analysis of the DEGs and DEPs, it was found that the two key KEGG pathways were mainly enriched—the PPAR signaling pathway and ferroptosis.

The DEGs mainly enriched the 9 GO terms in the transcriptome, which primarily mediated the metabolism of nutrients, the immune response, the cellular composition, and the integrin signaling of lambs under weaning stress. Transaminase activity and plasma lipoprotein particles are closely related to intestinal fat digestion and absorption, supported by a study of Yokoyama (21). Yokoyama concludes that the level of transaminase in the small intestine is positively correlated with the synthesis of enzyme proteins and the transport of fatty acids to intestinal cells. In addition, the hexose transmembrane transporter activity is closely related to the digestion and metabolism of glucose and other monosaccharides and the small molecule catabolic process. Its significantly enrichment indicated that the absorption and transportation of nutrients have changed significantly after separating from the ewe at weaning. At the same time, the leukocyte activation and chemotaxis reflected the changes in intestinal immunity in lambs.

Furthermore, the greatly enriched KEGG pathways mainly included the PPAR signaling pathway and ferroptosis. Notably, these pathways are closely related to immune response, cellular process, and nutrient metabolism, which indicated that weaning stress most likely disturbed these processes. For example, the solute carrier (SLC) group of membrane transporters is mainly distributed on the cell membrane (22). It is the main intestinal transporter of many amino acids and peptides (23). Meanwhile, according to Liao and Gaowa's study, it was found that changing the nutrient intake ratio of young ruminants would lead to an increased expression level of nutrient transporters in jejunal enterocytes (24, 25).

Although in this study, the correlation between the transcriptome and proteome of the same samples was low and it is a common phenomenon (26, 27). The main reasons for this phenomenon might be: (1) the process of transcription and translation is very complex and there are RNA-mediated transcription regulation and posttranslational protein modification, so transcriptome analysis cannot completely reveal protein expression and (2) both the RNA and protein quantitative methods have their limitations, such as: RNA-seq may involve in overestimation or underestimation of the expression level, while iTRAQ will show a significant underestimation of the degree of protein upregulation or downregulation (28). Therefore, in the recent years, comprehensive transcriptomic and proteomic methods have been conducted in diverse study fields. The integrating proteomic and transcriptomic analysis showed that the two GO terms (intestinal lipid absorption and urea cycle) and the 2 KEGG pathways (PPAR signaling pathway and ferroptosis) were significantly downregulated after weaning stress.

Peroxisome proliferator-activated receptors, belonging to the nuclear receptor family, are a lipid ligand-activated nuclear transcription factor, including three members: PPAR-γ, PPAR-α, and PPAR-β/δ (29), which play a vital role in growth and development, carbohydrate and lipid metabolism, immunity, and inflammation (30, 31). The main mechanism of the PPAR signaling pathway is that it binds to the peroxisome proliferator response element (PPRE) as a heterodimer with retinoid X receptor (RXR) after the PPAR activation to regulate the expression of the target gene, which is vital for the regulation of intestinal immune response and fat metabolism. The PPARs control intestinal immune response mainly by changing the phenotype of macrophages and combining with short-chain fatty acids to induce the expansion and differentiation of T lymphocytes (Treg cells) (32–35). Notably, butyric acid is the main carbon source of intestinal epithelial cells (36) and the ligand of the PPAR (33). In addition, if the PPAR-α pathway is inhibited, the expression of interleukin-22 (IL-22) secreted by Th1 and Th17 cells will decrease and the structural integrity of the intestinal mucosal barrier will be destroyed, resulting in an increased intestinal susceptibility to bacteria and endotoxin (37, 38). Therefore, the changes in milk replacer nutritional composition compared with ewe's milk may lead to the accumulation of intestinal microorganisms and their metabolites and the steady-state imbalance of lambs.

As shown in Figure 7B, *APOA2, APOC3, ACSL3, CPS1, FABP2*, and *PCK1* were significantly enriched in the PPAR signaling pathway. Among these, *PCK1* encodes a cytoplasmic enzyme in sheep intestines to promote the production of phosphoenolpyruvate (39) and control the production of glycerol and lipids (40), which plays an essential role in intestinal lipid metabolism. *FABP2* encodes an intestinal fatty acid-binding protein and is only distributed in mature intestinal cells. Hence, the downregulation of its expression indicates the decrease of intestinal lipid metabolism and the blocked intestinal development of lambs (41). *APOA2* mediates the activation of the PPAR signaling pathway in the intestine (42). Like *APOC3*, the proteins they encode are related to the metabolism of triglyceride-rich lipoproteins and there is a strong interaction between them (43, 44). Besides, *APOA2* is also involved in the metabolism of free fatty acids, but *APOC3* itself, such as lipopolysaccharide, can promote the release of interleukin-1β (IL-1β) from monocytes and induce inflammation. Several studies have shown that weaning, as the most severe stimulating factor in early life, can destroy intestinal mucosal barrier structure, reduce immune and antioxidant function, increase the expression of IL-1β and tumor necrosis factor-α (TNF-α), and induce intestinal inflammation (1, 5, 45). In summary, we speculated that weaning stress downregulated the expression of the PPAR signaling pathway, resulting in intestinal immune function maladjustment, the structural integrity of intestinal mucosal barrier destruction, increased susceptibility to endotoxin, and induced severe inflammatory response. Moreover, the downregulation of the PPAR signaling pathway led to the disorder of lipid metabolism and the decrease of the PPAR ligands, which further aggravated this process.

Ferroptosis is an iron-dependent regulatory form of cell death, different from apoptosis, necrosis, and autophagy, which involves fatal iron-catalyzed lipid damage (46). The main mechanism is that Fe^2+^/ester oxygenase catalyzes the high expression of polyunsaturated fatty acids (PUFAs) on the cell membrane, resulting in lipid peroxidation and reactive oxygen species (ROS) accumulating, further inducing cell death. In addition, PUFAs is more susceptible to lipid peroxidation (47) and free PUFAs inserted into extracellular phospholipids after esterification can be used as a signal of iron ptosis after oxidation (48). Previous studies have shown that the Acyl-CoA synthetase long-chain (ACSL) family is involved in PUFA-phosphatidylethanolamines (PUFA-PEs) biosynthesis in the cell membrane and is the substrate of lipid peroxidation (48). Interestingly, our combined transcriptome and proteome analysis showed that the expression of *ACSL3* was significantly downregulated. Above all, we speculated that after separating from the ewe at weaning, the lambs downregulated the expression of *ACSL3* and other relative genes to resist the intestinal injury caused by ROS, resulting in the remodeling of PUFAs on the cell membrane, the disorder of lipid metabolism and absorption, and the lack of lipid peroxidation substrate, thus enhancing the resistance to ferroptosis. Notably, it has been already found that the normal development of the human fetal immune system depends on adequate intake of PUFAs, so it may be related to ferroptosis, which provides a new idea for us to study the regulation of the intestinal immune system in weaned lambs (49). Above all, we infer that the cause of ferroptosis is the lack or the low concentration of transferrin in milk replacer, which results in the obstruction of absorption and transport of iron ions in the intestine and the accumulation of Fe^2+^ in the epithelial cells of the intestinal mucosa. This result is consistent with the exfoliation and death of epithelial cells observed in the previous section and the effects of epithelial cell proliferation and disturbance of nutrient absorption and transport in the transcriptional group.

The integrating proteomic and transcriptomic analysis also showed that immunoglobulin-mediated immune response, *Staphylococcus aureus* infection, and negative regulation of leukocyte differentiation were significantly upregulated in transcriptome, but downregulated in the proteome. Due to post-transcription regulation, there is a deviation between transcriptome analysis results and protein expression. Some previous studies have shown that microRNA (miRNA) mediates the regulation of cell response under stress (50, 51). It was found that miR-146b could directly act on the Toll-like receptor 4 (TLR4) of intestinal epithelial cells of piglets to regulate intestinal metabolism and immune response of piglets under weaning stress (52). Based on this, we speculate that miRNA-mediated post-transcriptional regulation of messenger RNA (mRNA) may be involved in regulating intestinal metabolism and immune response of lambs under the stress of separating from the ewe at weaning. Therefore, to reveal the molecular regulatory mechanism of intestinal injury of lambs under weaning stress, we will further explore miRNA and its target genes in our following study. In addition, this study has only been conducted for 15 days. The long-term effect of early weaning on intestinal gene expression in lambs is unknown, worthy of further exploration in future study.

## Conclusion

In this study, we used transcriptome and proteome to enrich and analyze the DEGs and found that the PPAR signaling pathway and ferroptosis may be the key drivers damaging the small intestine of lambs. Significantly differentially expressed genes such as *FABP2, ACSL3, APOA2, APOC3*, and *PCK1* may be the key genes. These results could provide new ideas for alleviating the intestinal injury of lambs caused by weaning ewe's milk and a theoretical basis for preparing the new and efficient milk replacer.

## Data Availability Statement

The datasets presented in this study can be found in online repositories. The names of the repository/repositories and accession number(s) can be found below: BioProject, Accession Number PRJNA781073.

## Ethics Statement

This trial was conducted at the Linqing Runlin Animal Husbandry Corporation Ltd., in Shandong, China. All the experiments were performed according to the Regulations for the Administration of Affairs Concerning Experimental Animals published by the Ministry of Science and Technology, China, in 2004. The Chinese Academy of Agricultural Sciences Animal Ethics Committee approved all the experiments and human animal care and handling procedures were followed throughout the experiment (AEC-CAAS-20160715).

## Author Contributions

LH contributed to study design, sample processing, data analyses, and writing the original draft. SW and DH contributed to data analyses, review, and editing. HT, LK, and QD contributed to the conception, study design, and sample collection. KC contributed to project administration, study design, and supervision. All authors contributed to writing the manuscript.

## Funding

The National Natural Science Foundation of China (32172764) and the Cooperative Guidance Project of Prospering Inner Mongolia Through Science and Technology in 2021 (2021CG0024) supported this study.

## Conflict of Interest

The authors declare that the research was conducted in the absence of any commercial or financial relationships that could be construed as a potential conflict of interest.

## Publisher's Note

All claims expressed in this article are solely those of the authors and do not necessarily represent those of their affiliated organizations, or those of the publisher, the editors and the reviewers. Any product that may be evaluated in this article, or claim that may be made by its manufacturer, is not guaranteed or endorsed by the publisher.
