# Supplementary material for: Transcriptome and iTRAQ-Based Proteome Reveal the Molecular Mechanism of Intestinal Injury Induced by Weaning Ewe's Milk in Lambs
Source: Front Vet Sci. 2022 Apr 25;9:809188. doi: 10.3389/fvets.2022.809188 (PMC9082421; doi:10.3389/fvets.2022.809188)
Supplement: Supplementary file 1 [file Image_1.pdf]

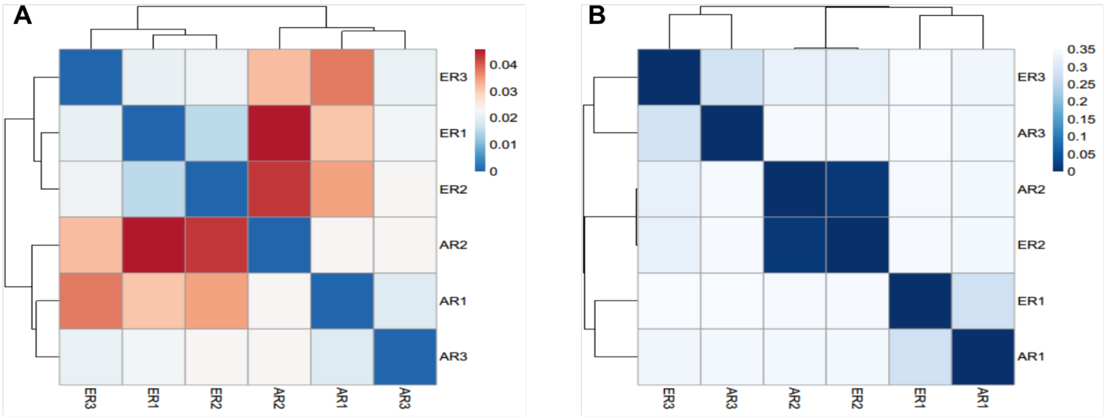

FIGURE S1 | The sample clustering based on the gene expression and SNPs. (A) Correlation matrix of 6 RNA-seq libraries. Pairwise Pearson correlation coefficients were calculated based on normalized gene read counts using DESeq2. Samples were hierarchically clustered with the Euclidean distance method. The color scale indicates the degree of correlation. (B) IBS distance matrix of 6 samples. The IBS matrix was estimated based on the SNPs of the RNA-seq reads. The color scale indicates the IBS distance between each pair of individuals.
